# The awareness of neglected tropical diseases in a sample of medical and nursing students in Cairo University, Egypt: A cross-sectional study

**DOI:** 10.1371/journal.pntd.0008826

**Published:** 2020-11-18

**Authors:** Eman Elfar, Noha Asem, Hanaa Yousof

**Affiliations:** Public Health and Community Medicine Department, Faculty of Medicine, Cairo University, Egypt; Federal University of Agriculture Abeokuta, NIGERIA

## Abstract

Neglected tropical diseases (NTDs) are a group of chronic diseases affecting 1.2 billion people worldwide, with more burden in the developing communities. Improving awareness about NTDs is a powerful affordable long-term intervention for infection control. In literature, there is a limited number of studies in the developing countries assessing the awareness of healthcare providers regarding these diseases. The present study aimed at assessing the awareness of a sample of Cairo University medical and nursing students regarding NTDs. A cross-sectional descriptive study was conducted on 184 medical and nursing students in Cairo University. An anonymous self-administered questionnaire in English language with an estimated completion time of 15 minutes was used for evaluation. It included question categories which cover the knowledge about NTDs and control measures as well as the willingness to participate in NTDs control activities. Content analysis was performed on the materials and specifications of the epidemiology course given to medical and nursing students. Out of the study participants, 26% knew the meaning of NTDs. The main source of their knowledge was social media followed by the epidemiology course. A percentage of 33% of the students agreed that NTDs are of public health importance in Egypt. Thirty four percent of the participants expressed their willingness to participate in control activities for NTDs. Comparing medical and nursing students, a higher percentage of the nursing students stated that NTDs are causing a public health problem in Egypt with a statistically significant difference (P value < 0.001), while a statistically significant higher percentage of medical students believed that the awareness level regarding NTDs in Egypt is low (P value = 0.002). Cairo University medical and nursing students in this study showed a gap in the level of knowledge regarding NTDs and their control activities which represents a great threat to the control of these diseases.

## Author summary

Egypt vision 2030 aiming that all Egyptians should have healthy, safe, and secure life. In the Middle East and North Africa region, Egypt has the highest rate of NTDs. Current medical and nursing students are the future healthcare workers who will be responsible to participate in control activities for the elimination of these diseases in Egypt. The present study aimed to provide information about the awareness of medical and nursing students regarding NTDs and their control activities. We targeted medical and nursing students who have finished an epidemiology course. Our results showed that only one fourth of the students knew the meaning of NTDs and nearly half of them agreed that the awareness of NTDs is poor among Egyptians. Although only 5% of the included students are aware of NTDs control activities, about one third of them agreed to participate in these activities. Nowadays, there is an urgent need to update the curricula of medical students in Egypt, especially because of the new program the medical schools in Egypt are experiencing currently (five years of studying and two years for training). Also, the engagement of medical and nursing students in research and prevention activities to combat NTDs should be encouraged in the training years.

## Introduction

Neglected tropical diseases (NTDs) are a group of infectious diseases which are categorized among the most common diseases worldwide, especially in the low-income societies suffering from deficiency of sanitation and healthcare services in Africa, Asia, and Latin America [1,2, 3]. The World Health Organization (WHO) estimated that NTDs affect 1.2 billion people worldwide and cause around 534,000 deaths every year [3,4]. Although NTDs vary in their distribution, epidemiology, impact, and control, they share common features such as being treatable or preventable through relatively simple public health interventions [4,5].

Egypt has the highest rates of NTDs in the Middle East and North Africa region; these diseases are endemic in Egypt since Pharaonic time [6]. NTDs include schistosomiasis, soil-transmitted helminthiasis, lymphatic filariasis, leishmaniasis, fascioliasis, and the epidermal parasitic skin diseases. Also, polyparasitism (multiple parasitic infections) is prevalent in Egypt and it magnifies the problem of NTDs by increasing the morbidity and susceptibility to other infections [6].

WHO targets and sets timelines for control and elimination of NTDs. The London Declaration, signed in 2012, established a road map to help nations reach the control or elimination of at least 10 NTDs by 2020 [7]. Combating NTDs is pivotal for achieving the third sustainable development goal (SDG 3) of ensuring healthy lives and promoting wellbeing for all at all ages [8]. Therefore, addressing NTDs elimination is important. The key objective of going forward with 2030 goals is finding optimal ways to effectively integrate inter- and intra-sectoral NTDs interventions and programs to be aligned with the sustainable development goals and universal health coverage (UHC). The first recommendation for integrating activities is to improve the policies based on the principles of UHC and broader health systems [8,9,10].

Despite the ongoing efforts to improve the socio-economic status, environmental sanitation, and ecology of the Egyptian community, much effort is still required [6]. An improvement in the tools diagnosing NTDs is needed to apply more sustainable effective treatment strategies at different stages of control, interruption of transmission, elimination, and post-elimination surveillance. Also, an increase in the public-private partnerships is needed to ensure that the progress made is consolidated and sustained for extensively strengthening health systems [11]. Therefore, the awareness of healthcare providers regarding NTDs must be improved to play a central role in the diagnosis, treatment, and control of these diseases [12].

The World Health Organization and the U.S. Agency for International Development (USAID) recommended integrated programs and approaches targeting NTDs control or elimination including public health actions, medications, ecological discipline, veterinary science, food safety, and economics [13,14].

Similarly, more resources are needed to monitor and evaluate the established and ongoing activities. There is a need to improve public awareness and engage people in advocacy in their country [8,15].

The knowledge of physicians and nurses about NTDs represents the cornerstone for the control of these diseases, especially in the developing countries. Thus, trainings covering these diseases should be included during medical and nursing education [16]. Medical and nursing students are the future healthcare workers who will be responsible to carry out the control activities to achieve elimination of NTDs in Egypt. Although it is essential to ensure that the existing curricula adopted in medical and nursing education in the developing countries fulfill the clinical and epidemiological needs of these diseases, there is a limited number of studies assessing the knowledge level of medical and nursing students regarding NTDs [17]. To the best of our knowledge, the studies assessing the knowledge level of medical and nursing students regarding NTDs in Egypt are scarce. Therefore, the objectives of this study were to assess the awareness of medical and nursing students regarding NTDs and identify their willingness to participate in the control activities targeting NTDs in Egypt.

## Methods

### Ethics statement

The approval of the Research Ethics Committee of Faculty of Medicine, Cairo University was sought (N-39-19) before the study conduction. The data collection was anonymous and hence, all responses were confidential. An oral consent was sought from all study participants before participation in the study and after explanation of the study’s aim as well as assurance of data confidentiality. Approvals were also obtained from the epidemiology course coordinator in the Faculty of Nursing, Cairo University and the head of public health department in the Faculty of Medicine, Cairo University before the study conduction. The Research Ethics Committee of Faculty of Medicine, Cairo University approved the study (N– 39–19).

The current study is a cross-sectional descriptive study, conducted on medical and nursing students from Faculty of Medicine and Faculty of Nursing (providing bachelor-degree in nursing), Cairo University. Both faculties are located within the same campus.

The study population included medical and nursing students of Cairo University who were enrolled in the epidemiology and biostatistics course, academic year 2018/2019. The study sample consists of two parts: the whole population sample including all the nursing students enrolled in the epidemiology course during the second semester of the academic year 2018/2019 and a convenient (non-random) sample including fourth year medical students who finished their epidemiology course. All nursing students (n = 110) who finished the epidemiology course during the second semester of the academic year 2018/2019 were invited to participate in this study. Also, an equal number (n = 110) of fourth year medical students who finished the epidemiology course were invited.

An anonymous self-administered questionnaire was distributed to the included students. This questionnaire was designed to evaluate the awareness of students regarding NTDs. It took 15 minutes to be filled. The questionnaire was designed in English language and included close-ended 3-point responses (yes, no, or do not know). It covered the following categories: knowledge about NTDs, knowledge about control measures, and willingness to participate in NTDs control activities.

The questionnaire was developed by the researchers after extensive literature review. In addition. It was revised by 3 different public health experts and changes were made accordingly.

Before the study implementation, a pilot test for the questionnaire form was done among 22 potential participants (10% of the expected sample size) to check the validity and clarity of the structured questionnaire and estimate the time needed to complete it. The data from this pilot test were not included in the analysis.

Content analysis on the medical and nursing epidemiology course materials and specifications was performed manually for the presence and frequency (qualitative and quantitative) of the phrase “neglected tropical disease” or the abbreviation “NTDs”. For content validity, the analysis was done by 3 public health staff members (the researchers) independently. The results of the analyses performed by the researchers were 100% consistent.

### Data analysis

Data were entered and coded using Microsoft Office Excel 2010. Statistical analysis was done using IBM SPSS version 24 (IBM Corporation, USA, Armonk, New York, 2016). Frequencies (number) and relative frequencies (percent) were used to summarize qualitative variables, while mean and standard deviations were used to summarize quantitative variables. The comparison between groups was done using chi-square test. P value less than or equal to 0.05 was considered significant.

## Results

A total of 184 students (106 students from Faculty of Medicine and 78 students from Faculty of Nursing) completed the questionnaire with overall response rate of 87.6%. The age of the included students ranged from 20 to 25 years with mean and SD of 22 ± 1.2 years. Data were collected at the end of the epidemiology course for both faculties in the academic year 2018/2019.

The epidemiology of communicable diseases course for medical and nursing students is divided into general epidemiology (infectious cycle, pattern of disease occurrence, and general measures of prevention and control of communicable diseases) and specific epidemiology. The specific epidemiology of infectious diseases is divided according to the 4 main modes of transmission, namely: droplet, foodborne, contact, and arthropod borne infections. Most of the NTDs are included in the course such as schistosomiasis, lymphatic filariasis, leishmaniasis, rabies, soil transmitted helminthiasis (STH) as ascariasis and hookworm infections. Neither the term “neglected tropical disease” nor the abbreviation “NTDs” was mentioned explicitly in the course materials or specifications.

Nearly equal proportions of males and females were included in this study; more than three fourths of them are Egyptians (Table 1). Only one fourth (26%) of the students knew the meaning of NTDs; the main source of their knowledge is social media, followed by epidemiology course and mass media (TV/Radio) (Table 2). The epidemiology course was mentioned as a single source of knowledge by 8 students (4.3%), while it was mentioned with other sources by 6 students (3.3%); 4 of them mentioned it with mass media (TV/Radio) and social media.

**Table 1:** Basic characteristics of the included medical and nursing students in Cairo University.

Variable		Number (Total = 184)	%
Sex	Male	84	45.7
	Female	100	54.3
Faculty	Medicine	106	57.6
	Nursing	78	42.4
Academic level	Second	4	2.2
	Third	74	40.2
	Fourth	106	57.6
Nationality	Egyptian	150	81.5
	Non-Egyptian	34	18.5
Previous year grade	Excellent	26	14.1
	Very good	78	42.4
	Good	50	27.2
	Pass	30	16.3

**Table 2:** Knowledge of the included medical and nursing students in Cairo University regarding NTDs.

Question		Number (Total = 184)	%
Know the meaning of NTDs?	Yes	48	26.1
	No	100	54.3
	Not sure	36	19.6
Heard of NTDs in Egypt?	Yes	26	14.1
	No	104	56.5
	Not sure	54	29.3
Where did you hear about NTDs? *			
Scientific journal	yes	8	4.3
Faculty library	yes	4	2.2
Social media	yes	16	8.7
Epidemiology course	yes	14	7.6
TV/Radio	yes	14	7.6
Conferences/ meetings	yes	6	3.3
Recognition of different NTDs by the included medical and nursing students			
NTDs constitute a problem of Public health importance in Egypt?	Yes	60	32.6
	No	40	21.7
	Not sure	84	45.7
Have you ever seen someone affected by NTD?	Yes	18	9.8
	No	88	47.8
	Not sure	78	42.4
Which of the following is NTD? *			
Schistosomiasis		26	14.1
Onchocerciasis		10	5.4
Leprosy		22	12.0
Trachoma		20	10.9
Buruli ulcer		10	5.4
Leishmaniasis		24	13.0
Rabies		8	4.3
Human African Trypanosomiasis		44	23.9
Lymphatic Filariasis		26	14.1
Dracunculiasis		14	7.6
Chagas disease		20	10.9
Soil transmitted helminthiasis		30	16.3

* more than one answer allowed

One third of the included students (33%) agreed that NTDs are causing a problem of public health importance in Egypt, while only 9% stated that they had seen a case of NTDs. Low percentage of the studied medical and nursing students (ranged from 4.3 to 23.9%) correctly identified the names of individual NTDs (Table 2). Very low percentage of the students (5%) knew specific activities for NTDs prevention and control in Egypt, while more than one third of them (34%) expressed their willingness to participate in these control activities. Nearly half of the students agreed on the poor level of awareness regarding NTDs in Egypt (Table 3).

**Table 3:** Knowledge regarding the prevention and control of NTDs among the included medical and nursing students of Cairo University.

Variable		Number (Total = 184)	%
Do you know about specific NTDs control activities?	Yes	10	5.4
	No	114	62.0
	Not sure	60	32.6
Are you willing to participate in control activities for NTDs?	Yes	62	33.7
	No	70	38.0
	Not sure	52	28.3
Do you think that awareness of NTDs is poor in Egypt?	Yes	90	48.9
	No	36	19.6
	Not sure	58	31.5

On comparing medical and nursing students, no significant difference (P value = 0.928) was found in their knowledge about NTDs (Table 4). However, higher percentage of nursing students stated that NTDs are of public health significance in Egypt compared to medical students (P < 0.001). A statistically significant higher percentage of medical students believed that the awareness level of NTDs in Egypt is low (P value = 0.002). No statistically significant difference (P value = 0.061) was found between medical and nursing students regarding their willingness to participate in the control activities for NTDs (Table 4).

**Table 4:** Comparison between the knowledge of the included medical and nursing students about NTDs in Cairo University.

Variable		Faculty				P value
		Medicine (Total = 106)		Nursing (Total = 78)		
		Number	%	Number	%	
Know the meaning of NTDs	Yes	28	26.4	20	25.6	0.928
	No	56	52.8	44	56.4	
	Not sure	22	20.8	14	17.9	
Heard of NTDs in Egypt	Yes	10	9.4	16	20.5	0.239
	No	60	56.6	44	56.4	
	Not sure	36	34.0	18	23.1	
Where did you hear of NTDs? *	Scientific journal	2	1.9	6	7.7	0.203
	Faculty library	0	0.0	4	5.1	0.177
	Social media	4	3.8	12	15.4	0.067
	Epidemiology course	4	3.8	10	12.8	0.106
	TV/Radio	6	5.7	8	10.3	0.411
	Conferences/ meetings	2	1.9	4	5.1	0.387
NTDs constitute a problem of public health importance in Egypt?	Yes	28	26.4	32	41.0	<0.001
	No	12	11.3	28	35.9	
	Not sure	66	62.3	18	23.1	
Have you ever seen someone affected by NTDs?	Yes	10	9.4	8	10.3	0.016
	No	38	35.8	50	64.1	
	Not sure	58	54.7	20	25.6	
Do you know about specific NTDs control activities?	Yes	4	3.8	6	7.7	0.034
	No	56	52.8	58	74.4	
	Not sure	46	43.4	14	17.9	
Are you willing to participate in control activities for NTDs?	Yes	32	30.2	30	38.5	0.061
	No	34	32.1	36	46.2	
	Not sure	40	37.7	12	15.4	
Do you think that awareness to NTDs is poor in Egypt?	Yes	56	52.8	34	43.6	0.002
	No	8	7.5	28	35.9	
	Not sure	42	39.6	16	20.5	

* more than one answer allowed

## Discussion

One of Egypt vision 2030 aims is that all Egyptians should have healthy, safe, and secure life [18]. NTDs represent a major public health problem in Egypt [6]. Therefore, combating NTDs is crucial to achieve the 2030 vision target. Raising awareness about NTDs can serve as a powerful affordable intervention for infection control. Also, the high level of awareness is capable of achieving significant and long-lasting results in controlling these diseases [18,19]. There are only few studies in literature assessing the knowledge of medical and nursing students about NTDs; especially in the developing countries [17].

This cross-sectional study was carried out to assess the level of awareness of Cairo University medical and nursing students regarding NTDs. Also, it addressed their knowledge gap as well as their perceived role in diagnosis, treatment, and control of NTDs presently and in the future. This is premised on the belief that the knowledge about NTDs should be started and upscaled during medical and nursing school training [12,17]. The results of awareness assessment in this study underline the need for structured teaching programs for medical and nursing students in the context of NTDs and their preventive strategies. This study has shown that about one fourth of the included students knew the meaning of NTDs; a low percentage ranging from 4.3 to 23.9% correctly identified the names of individual NTDs and a very low percentage of the total included students (5%) knew specific activities for prevention and control of NTDs in Egypt. The gap of epidemiological knowledge about NTDs is consistent with the content analysis of the epidemiology course which indicates that the term “NTDs” was not mentioned explicitly in the course materials or specification. Moreover, only (4.3%) of the included students mentioned the epidemiology course as a single source of knowledge about NTDs.

Although schistosomiasis and lymphatic filariasis are the most common NTDs in Egypt [6], about 15% only of the included students could identify them as NTDs. It is alarming to know that all the participants finished their epidemiology course with meager epidemiological knowledge about endemic NTDs in Egypt. A study by Villafuerte-Galvez J.et.al 2008 conducted in Peru including medical students from all grades revealed that the students’ epidemiological knowledge about NTDs remained the same for the rest of studying years, while their clinical practice improved by actively participating in training, research, and publication to fight NTDs in their country [20].

In the current study, we found that one third of the included medical and nursing students agreed that NTDs are causing a problem of public health importance in Egypt. This could represent a major challenge to control NTDs as community awareness is usually carried out by community nurses and healthcare workers [21]. Also, many studies stated that healthcare workers should be aware of community diseases [4,22].

In the current study, the students themselves perceived a gap in the public awareness regarding NTDs where nearly half of them agreed on the low level of public awareness regarding these diseases in Egypt. On the other hand, a study done in Nigeria to assess the general citizen’s knowledge and awareness of NTDs control activities found that a significant proportion of the included subjects (73.1%) have heard of NTDs before, yet only 63.1% have good knowledge about them [23]. Lemoine and colleagues stated that the adherence to management guidelines was positively associated with the population’s awareness and understanding of the disease, its modes of transmission, mass drug administration campaign, expected adverse effects, and the subject's perception about the risk of infection [24].

Although the Ministry of Health in Egypt applies control programs for NTDs by offering anti-helminthic drugs free of charge and deworming campaign [25], this study showed that only 5% of the included medical and nursing students knew specific activities for prevention and control of these disease in Egypt. This may be attributed to the fact that specific prevention and control measures for NTDs and the efforts exerted nationally or globally to combat them are not covered properly in the epidemiology course they studied. Also, this could reflect poor advertising of these programs in TV/radio and social media when compared to other control programs like the hepatitis C virus (HCV) control program where only 8.7% of the students heard about NTDs from social media and 7.6% heard about them about NTDs from TV/radio. Hence, more edification and enlightenment on the activities of governorates regarding the control of NTDs is suggested.

The results of the present study showed that about two thirds of the students were unwilling (38%) or not sure (28%) to participate in the control activities for NTDs. Previous studies showed that the increased knowledge of students about epidemiology, prevention, and control activities for NTDs can help the students to make informed decisions regarding participation in NTDs control activities and influence their refusal to participate in these activities [19]. One third of the included medical students were willing to participate in NTDs control activities; this represents a great opportunity that should be invested by the faculty. Resources and fund should be allocated for the control activities for NTDs. Many studies worldwide recommended that medical students should be encouraged to perform community research studies with university financial support for research and join international training programs focusing on field research activities and mutual good chances between the developed and developing countries [22,26,27].

### Limitations

The results of studies of knowledge assessment are descriptive and do not explain the reasons for these findings. Thus, the researchers cautiously interpreted the results of this study. Also, the researchers faced difficulties in conducting FGDs due to the constraints of gathering students during the period of SARS-CoV-2 pandemic lockdown. The absence of a former study to tackle this important topic in medical and nursing students in Cairo or Egypt represented an obstacle to compare our results appropriately. However, this study addresses the importance of assessing the knowledge of medical and nursing students regarding NTDs. Furthermore, our findings afford useful information which future researchers can depend on for further development of structured research and teaching strategies.

### Conclusion and recommendation

There is a gap in the knowledge of medical and nursing students of Cairo University regarding NTDs and their control activities which represents a great challenge to the control of NTDs. Learning about NTDs is essential for medical and nursing students. Hence, Egyptian medical and nursing curricula should be updated. NTDs concepts, epidemiology, and management in detail must be integrated in the medical and nursing curricula and practical training. Faculties of medicine and nursing should offer more opportunities to promote the engagement of students in research and training activities on NTDs. Continuous assessment of ongoing activities in training and research is recommended to get use of the structured medical and nursing students' clusters. Repeating the study on a nationally representative sample of medical and nursing students in all grades in Egypt is recommended using focus group discussions and personal interviews.

## Supporting information

S1 Data Study data. (XLSX)
